# Incidence of Acute Endophthalmitis After Intravitreal Bevacizumab Injection at a Tertiary Care Hospital in Lahore

**DOI:** 10.7759/cureus.13185

**Published:** 2021-02-06

**Authors:** Nasir Ahmed, Hafeez Ur Rehman, Memoona Rafique, Muhammad S Hamza, Huma A Mirza

**Affiliations:** 1 Department of Ophthalmology, King Edward Medical Univeristy, Mayo Hospital, Lahore, PAK; 2 Department of Ophthalmology, King Edward Medical University, Mayo Hospital, Lahore, PAK

**Keywords:** acute endophthalmitis, bevacizumab, intravitreal, incidence, ophthalmology

## Abstract

Objective: We aimed to assess the incidence, management, and visual outcome of acute endophthalmitis in patients following intravitreal bevacizumab injection in a tertiary care setup. It was a prospective and single-center database study.

Patients and methods: Patients receiving intravitreal bevacizumab injections for various retinal vascular diseases from January 2019 to September 2020. The study was carried out at the Institute of Ophthalmology, Mayo Hospital, Lahore over a period of 21 months. Preformed bevacizumab injections were administered intravitreally on patients of various retinal vascular diseases under strict aseptic measures and by following the standard guidelines. The patients were put on follow-ups for a duration of four weeks to see any signs of acute endophthalmitis.

Results: A total of 3051 injections were administered in 1104 eyes of 743 patients during the above-mentioned study period. The incidence of endophthalmitis was found to be 0.0328% (1/3051). The patient, who developed endophthalmitis, was treated with topical and intravitreal antibiotics followed by vitrectomy that resulted in clinically significant improvement in vision.

Conclusion: Incidence of acute endophthalmitis following intravitreal bevacizumab injection was low and could be prevented by taking strict aseptic measures during administration.

## Introduction

Bevacizumab is a monoclonal antibody that inhibits vascular endothelial growth factor-A (VEGF-A). It is primarily an anti-cancerous drug approved to be used systematically in a variety of cancers like colorectal, renal, etc. It works by blocking the abnormal proliferation of blood vessels by inhibiting VEGF-A, so it is also being employed in various ocular vascular proliferative disorders, thus preventing and slowing down visual loss caused by leakage from the newly formed vessels. It is indicated in retinal vascular diseases like exudative age-related macular degeneration (ARMD), or macular edema due to diabetic retinopathy and retinal vein occlusion. It is administered intravitreally in an operation theatre setup. This intravitreal route of administration is considered to be relatively safe, however, it can cause many ocular and systemic complications like endophthalmitis, retinal detachment, cataract formation, stroke, increase in blood pressure, etc. None of these adverse events has a rate exceeding 0.27% [1].

Among these complications, the most serious and sight-threatening is acute endophthalmitis. This complication has a very low incidence (0.02-0.08%) of occurrence globally, but the prognosis is very poor [1,2]. Once it develops, the patients have clinically shown reduced vision, pain, and redness in the affected eye with hypopyon and lid & corneal edema as the prominent signs [3]. The post-injection endophthalmitis can be infectious or non-infectious [4].

With the increasing rate of retinal diseases, the frequency of this off-label intravitreal injection is also increasing exponentially worldwide hence exposing the population to this serious sight-threatening complication. Therefore, strict measures for its prevention should be given due importance. The factors that have been found in various retrospective studies to be responsible for increasing its risk include suppressed immunity (e.g., due to diabetes mellitus), abnormalities of the ocular surface, lack of aseptic technique of administration, and poor wound management [5]. On the other hand, improvement in administration technique, education of the patient about the symptoms of endophthalmitis, and maintenance of sterile conditions during administration are effective in controlling the incidence of endophthalmitis [6]. Retinal vascular diseases develop bilaterally in both eyes in the majority of patients. The administration of injections bilaterally on the same day is usually avoided by the surgeons due to associated risks and complications.

The purpose of this study is to assess the incidence, management, and visual outcome of acute endophthalmitis following intravitreal bevacizumab injection in patients coming to the Ophthalmology Department of Mayo Hospital, Lahore.

## Materials and methods

This was a prospective study carried out at the Institute of Ophthalmology, Mayo Hospital, Lahore. It included the patients who were administered injection bevacizumab (1.25 mg/0.05 ml) intravitreally for various retinal conditions, from January 2019 to September 2020. Informed consent was taken from all the patients and only those patients were included in the study who gave consent voluntarily. None of the participants were given any financial support for participation. All the eyes that had undergone any other intra-ocular procedure (e.g., cataract extraction) at least four weeks before and after the administration of injection were excluded from the study. Demographic data and past ocular & medical histories were recorded directly by interviewing the patients. A complete anterior and posterior segment examination along with B-scan and optical coherence tomography of the macula (where required) was carried out in all these patients before the prescription of injection.

All the bevacizumab injections were obtained from Shaukat Khanum Memorial Hospital, Lahore in the form of single-use prefilled sterile (29-gauge) syringes. These injections were administered by vitreoretinal consultants in an operation theatre setup of the Institute of Ophthalmology, Mayo Hospital, Lahore under strict aseptic measures. Proper surgical scrubbing was done before the administration of the injection. Wearing of sterile surgical gloves, surgical gown, face mask, and head cap throughout the procedure was ensured by all the surgeons. After taking proper consent from each patient, the topical anesthesia was given using 0.5% w/v proparacaine drops. Eyes to be injected were cleaned five minutes prior to injection by using 10% povidone-iodine solution. Later, sterile draping was done and a lid speculum was applied. The injection was administered preferably in the superotemporal quadrant of the sclera, 3.5 mm and 4.0 mm away from the limbus in pseudophakic and phakic eyes, respectively. These measurements were done with the help of sterile vernier calipers. After pulling out the needle, the entry site was held for some time with the help of conjunctival forceps to prevent regurgitation. A sterile eye pad was then applied to the eye and the patient was advised to remove it after four hours. Each patient was prescribed topical 'moxifloxacin' one drop four hours for one week [7]. A weekly follow up of up to four weeks was maintained for every patient. The patients were advised to present immediately in case of the development of any symptoms of endophthalmitis.

Acute post-injection endophthalmitis was diagnosed by its clinical signs and symptoms like pain, redness, acute visual loss, hypopyon, iritis, and vitritis within four weeks following administration of the injection. Any patient presenting with these clinical features within the defined time period was admitted immediately and was treated for acute endophthalmitis.

Statistical analysis was done using IBM Statistical Package for Social Sciences (SPSS) statistics version 25.0 (IBM, USA). The study was approved by the Institutional Review Board and Ethics Committee of the Institute of Ophthalmology, Mayo Hospital, Lahore.

## Results

A total of 743 patients were given intravitreal bevacizumab injections during the above-mentioned study period; 547 of these were males and 196 were females. The total numbers of injections were 3051, out of which 1947 were re-administered. These 3051 injections were administered on a total of 1104 eyes. Out of various indications for these injections, diabetic retinopathy was the most common (74%) followed by exudative age-related macular degeneration (15.5%) (Table 1). Most of the patients (60%) had aged in the range of 51-60 years. History of diabetes was positive in 89% of patients.

**Table 1:** Indications for Intravitreal Bevacizumab Injection

Indications for Injection Bevacizumab	Number of Patients	Percentage
Diabetic Retinopathy	552	74.3
Hypertensive Retinopathy	03	0.4
Vein Occlusion	52	7.0
Vitreous Hemorrhage	14	1.9
Neovascular Glaucoma	02	0.3
Retinal Detachment	05	0.7
Age-Related Macular Degeneration	115	15.5
Total	743	100.0

Out of these 3051 injections, one injection resulted in the development of acute endophthalmitis. Thus, the incidence of acute endophthalmitis after intravitreal injection of bevacizumab was 0.0328% (in 21 months) and per year rate was 0.018%. The rate of infection per eye was 0.09%.

The patient, who developed endophthalmitis after intravitreal bevacizumab injection, was a 49 years old male and presented two days after the administration of injection with complaints of ocular pain, redness, watering, and acute decrease of vision in his left eye. Visual acuity in the involved eye decreased from counting fingers to the perception of light (with normal projection in all quadrants). Clinical findings on detailed examination were conjunctival congestion, corneal endothelial dusting (prominent inferiorly), Grade-II anterior chamber reaction, and altered fundal glow with no fundal view. A B-scan of the left eye on the day of the presentation showed minimal intragel hemorrhage and diffuse choroidal edema. The patient was a known case of diabetes for the past six years. Indication of injection in this patient was choroidal vasculitis and vitreous hemorrhage in the left eye. There was no previous history of any ocular procedure in both eyes.

Management of endophthalmitis in this patient involved topical fortified vancomycin and ceftazidime drops and intravitreal injections of vancomycin (2 mg/0.1 ml) and ceftazidime (2 mg/0.1 ml) followed by "three ports pars plana vitrectomy (3PPPV)." Symptoms improved significantly after this intervention and his visual acuity in the involved eye at the time of discharge was ‘counting fingers’ and it improved to 6/36 after two months of vitrectomy.

## Discussion

Bevacizumab is an FDA authorized drug that is very effective in inhibiting angiogenesis in vision-threatening retinal vascular diseases [8,9]. Its side effects both local and systemic were studied in a number of previous researches [10]. Considering its intravitreal route of administration in ophthalmology, it can cause many complications [11]. Among these, the endophthalmitis is the significant one.

Incidence of endophthalmitis following the intravitreal bevacizumab injection varies from 0.02-0.08% [1,12,13]. In our study, the incidence of endophthalmitis is 0.038% and it is comparable to previous studies carried out at national and international levels. A database study carried out in Australia, New Zealand, and Switzerland by Daien et al. (2017) rate per injection was 0.012% (1/8013) [4]. The study also showed that the incidence of endophthalmitis increased with the increase in the total number of injections in an eye.

Sangroongruangsri et al. (2019) studied the incidence of endophthalmitis along with systemic events in patients from eight different hospitals in Thailand using a prospective study design. Patients were followed for six months and the rate of endophthalmitis was found to be less than 0.04% [1].

Haider et al. (2017) carried out an office-based study in Lahore to find out the incidence of acute endophthalmitis. Intravitreal bevacizumab injection was administered for various retinal conditions and 0.19% of the patients were clinically diagnosed with post-injection endophthalmitis [12].

Wani et al. (2016) studied the main indications for injection bevacizumab besides the incidence of post bevacizumab endophthalmitis at two different centers in Kuwait [13]. In their data collected, about 80% of the patients were having diabetic retinopathy. In our study as well, diabetic retinopathy is the major indication for the administration of bevacizumab injection (74.3%). The incidence of endophthalmitis in their study was 0.09%.

In our study, factors that are associated with increased risk of endophthalmitis like diabetes and any recent ocular surgery are studied. Many studies have successfully developed the association between the incidence of post-injection endophthalmitis and these risk factors [14]. In our study, the patient who developed endophthalmitis was a known case of diabetes for the past six years.

In our setting, prefilled single-use syringes were injected. These prefilled single-use syringes are proven to play an important role in reducing the incidence of post-injection endophthalmitis [15,16].

The prognosis of endophthalmitis is poor. It can progress to complete visual loss. Treatment options available for it, once it gets developed, is the intravitreal injection of antibiotics, followed by vitrectomy or evisceration [12,17,18]. The presented case in our study was treated with intravitreal injections of vancomycin and ceftazidime followed by 3PPPV and his visual outcome improved significantly after this intervention.

## Conclusions

In this prospective study, a total of 3051 injections were administered intravitreally in 1104 eyes, with a 0.0328% rate of post-procedure acute endophthalmitis which is very low and comparable to other studies. It is a serious complication that needs to be prevented by strictly following international protocols of administration of Intravitreal injection.
